# COVID-19 pandemic increased ESKAPEEc bloodstream infections and amplified carbapenem resistance in Chinese children: a multicenter surveillance study (2016–2023)

**DOI:** 10.3389/fcimb.2025.1685606

**Published:** 2025-10-20

**Authors:** Xiaoqiang Li, Hongmei Xu, Cai Wang, Ruiqiu Zhao, Jiaying Wu, Hui Yu, Chuanqing Wang, Pan Fu, Jikui Deng, Chunzhen Hua, Yinghu Chen, Mingming Zhou, Ting Zhang, Hong Zhang, Yiping Chen, Shifu Wang, Qing Cao, Huiling Deng, Huijun Cai, Jianhua Hao, Yuyang Zhou, Chunmei Jing

**Affiliations:** ^1^ Department of Clinical Laboratory, Children’s Hospital of Chongqing Medical University; National Clinical Research Center for Child Health and Disorders; Ministry of Education Key Laboratory of Child Development and Disorders. Chongqing Key Laboratory of Child Rare Diseases in Infection and Immunity, Chongqing, China; ^2^ Infectious Disease Department, Children’s Hospital of Chongqing Medical University; National Clinical Research Center for Child Health and Disorders; Ministry of Education Key Laboratory of Child Development and Disorders. Chongqing Key Laboratory of Child Rare Diseases in Infection and Immunity, Chongqing, China; ^3^ Infectious Disease Department, Children’s Hospital of Fudan University, National Children’s Medical Center, Shanghai, China; ^4^ Department of Clinical Microbiology Laboratory, Nosocomial Infection Control Department, Children’s Hospital of Fudan University, National Children’s Medical Center, Shanghai, China; ^5^ Infectious Disease Department, Shenzhen Children’s Hospital, Shenzhen, China; ^6^ Infectious Disease Department, Children’s Hospital of Zhejiang University, Hangzhou, China; ^7^ Department of Medical Laboratory, Children’s Hospital of Zhejiang University, Hangzhou, China; ^8^ Digestive and Infectious Disease Department, Children’s Hospital of Shanghai Jiaotong University, Shanghai, China; ^9^ Department of Medical Laboratory, Children’s Hospital of Shanghai Jiaotong University, Shanghai, China; ^10^ Pediatric Infectious Disease Department, Second Affiliated Hospital, and Yuying Children’s Hospital of Wenzhou Medical University, Wenzhou, China; ^11^ Department of Medical Laboratory, Qilu Children’s Hospital, Shandong University, Jinan, China; ^12^ Infectious Disease Department, Shanghai Children’s Medical Center, Shanghai, China; ^13^ Infectious Disease Department, Xi’an Children’s Hospital, Xi’an, China; ^14^ Department of Medical Laboratory, Xi’an Children’s Hospital, Xi’an, China; ^15^ Infectious Disease Department, Children’s Hospital of Kaifeng City, Kaifeng, China; ^16^ Department of Medical Laboratory, Children’s Hospital of Kaifeng City, Kaifeng, China

**Keywords:** bloodstream infections, ESKAPEEc, antimicrobial resistance, time trend, drift, neonatal, pediatric

## Abstract

**Objective:**

This study investigated the long-term trends in the distribution and antibiograms of ESKAPEEc pathogens in neonatal and pediatric bloodstream infections (BSIs), shifts in minimum inhibitory concentration (MIC) of vancomycin and linezolid in *Staphylococcus aureus*, along with the changing patterns of antimicrobial resistance phenotypes over time in China. This work provides a reference for the prevention and treatment of pediatric BSIs.

**Methods:**

A multicenter retrospective surveillance study was carried out from 2016 to 2023 at 12 tertiary pediatric hospitals across nine provinces and autonomous regions in China. The collected data were analyzed using GraphPad Prism 8 and WHONET 5.6. Temporal variations and linear trends were evaluated using chi-square or Fisher’s exact tests.

**Results:**

A total of 10,051 ESKAPEEc strains accounted for 22.5% (10,051/44,675) of all BSIs, with 32.3% from neonatal BSIs and 67.7% from pediatric BSIs. The detection rate of ESKAPEEc pathogens increased for post the coronavirus disease 2019 (COVID-19) compared to the pre-COVID-19. Carbapenem resistance levels were 5.5% in *Escherichia coli*, 28.0% in *Klebsiella pneumoniae*, 16.0% in *Enterobacter cloacae*, 12.5% in *Pseudomonas aeruginosa* and 38.5% in *Acinetobacter baumannii*. Both *Staphylococcus aureus* and *Enterococcus faecium* remained fully susceptible to vancomycin and linezolid. Between 2016–2019 and 2020–2023, resistance to ceftazidime and gentamicin decreased in *Escherichia coli* and *Klebsiella pneumoniae* while resistance to imipenem and meropenem increased. *Acinetobacter baumannii* exhibited reduced resistance to most antibiotics except cefotaxime, levofloxacin and amikacin. *Staphylococcus aureus* displayed a declining resistance to macrolides and aminoglycosides but increasing resistance to fluoroquinolones, whereas *Enterococcus faecium* exhibited reduced resistance to all tested antibiotics. Compared to neonatal BSIs, *Klebsiella pneumoniae* from pediatric BSIs exhibited lower resistance to all β-lactams especially carbapenems (32.3% vs. 15.0%) while *Acinetobacter baumannii* displayed higher resistance to all tested agents. Methicillin-susceptible *Staphylococcus aureus* (MSSA) strains had lower vancomycin MIC ≥2 μg/mL levels compared to methicillin-resistant *Staphylococcus aureus* (MRSA) strains. Significant temporal differences were observed in MRSA isolates with linezolid MIC ≥2 μg/mL but not in MSSA isolates. The MIC_50_ of vancomycin in MRSA strains was either equal to or higher than in MSSA strains from 2016 to 2022. The most frequently detected bacteria in carbapenem resistance, extended-spectrum cephalosporin resistance, fluoroquinolone resistance and aminoglycoside resistance were *Acinetobacter baumannii*, *Klebsiella pneumoniae*, *Escherichia coli* and *Acinetobacter baumannii* respectively.

**Conclusions:**

The incidence of ESKAPEEc in BSIs has increased, and the rising resistance to imipenem and meropenem in *Escherichia coli* and *Klebsiella pneumoniae* underscores the need for continued surveillance. Carbapenems remain effective against Gram-negative ESKAPEEc, while vancomycin and linezolid remain effective against Gram-positive ESKAPEEc. Age-stratified strategies are essential to manage carbapenem-resistant *Klebsiella pneumoniae* in neonatal BSIs and carbapenem-resistant *Acinetobacter baumannii* in pediatric BSIs. The MIC values for vancomycin in MRSA strains remained stable over time, whereas a decreasing susceptibility trend to vancomycin in MSSA strains and linezolid MIC shifts were not observed. Our findings are expected to provide to treatment of bloodstream infections in children and evidence on best practices and resource sharing for policy consideration to healthcare providers at the local and international levels.

## Introduction

Bloodstream infections (BSIs) can result in life-threatening conditions that include septicemia and remain a major cause of hospitalization and mortality for children, including neonates (Kontula et al., 2018; Lindell et al., 2020). The global BSIs incidence has risen significantly due to increased invasive procedures and widespread use of immunosuppressive agents (Kern and Rieg, 2020; Yang et al., 2023). Organ development and immune functions in children are not yet fully developed and thus make children particularly susceptible to BSIs. Pediatric BSI incidence has recently risen from 1.4 to 5.0% with accompanying mortalities as high as 16%, highlighting the urgency of addressing this condition (Folgori et al., 2014). The emergence of multidrug-resistant (MDR) bacteria further complicates clinical management and poses significant challenges for antimicrobial therapy (Yang et al., 2019). Accurate pathogen identification and early administration of appropriate antibiotics are critical for improving BSIs outcomes (Niederman et al., 2021; Yadav and Yadav, 2022). In particular, blood cultures continue to be the gold standard for BSI pathogen identification and antimicrobial susceptibility testing. These processes are crucial for determining appropriate treatment and preventing the rise of MDR bacteria (Kargaltseva et al., 2022; Péan de Ponfilly et al., 2021).

The ESKAPEEc group of highly virulent pathogens include Enterococcus faecium(E. faecium), Staphylococcus aureus(S. aureus), Klebsiella pneumoniae(K. pneumoniae), Acinetobacter baumannii(A. baumannii), Pseudomonas aeruginosa(P. aeruginosa), Enterobacter spp and Escherichia coli(E. coli), which are responsible for 50-70% of BSIs worldwide (Buetti et al., 2016; De Angelis et al., 2018; Gupta et al., 2024; Miller and Arias, 2024; Xu et al., 2016). The incidence of ESKAPEEc-related BSIs is rising rapidly (Pogue et al., 2015) and these infections are associated with severe consequences including prolonged hospital stays, higher healthcare costs and increased mortality (Founou et al., 2017; Marturano and Lowery, 2019; Zhen et al., 2019). Additionally, antimicrobial resistance of ESKAPEEc pathogens has further limited therapeutic options. However, global antimicrobial stewardship initiatives and vaccination programs have resulted in decreased distribution and resistance profiles of ESKAPEEc pathogens (Hu et al., 2016; Musicha et al., 2017). While epidemiological data for ESKAPEEc are available, most studies have focused on adult populations with limited emphasis on pediatric BSIs (Kritsotakis et al., 2022; Qin et al., 2024). Additionally, there is a lack of data on temporal shifts in resistance patterns among pediatric ESKAPEEc isolates in China.

To fill this gap, we carried out an 8-year multicenter retrospective study (2016–2023) aimed at investigating the changes in the composition and antimicrobial resistance profiles of ESKAPEEc pathogens in pediatric BSIs across China. Furthermore, we compared the distribution and antimicrobial resistance patterns of ESKAPEEc isolates between the pre-COVID-19 (2016–2019) and post-COVID-19 (2020–2023) periods.

## Materials and methods

### Patients and enrollment

Data on ESKAPEEc pathogens were collected from the Infectious Disease Surveillance of Pediatric (ISPED) database between 2016 and 2023. ISPED is a surveillance system designed to monitor pediatric infectious pathogens and resistance patterns in China. We utilized 12 tertiary children’s hospitals across nine provinces or autonomous cities: Chongqing, Jiangsu, Guangdong, Zhejiang, Shanxi, Shandong, Jilin, Henan and Shanghai. This study included only ESKAPEEc isolates from children with blood culture-confirmed BSIs in hospitals participating in ISPED. To assess trends in antimicrobial resistance (AMR) and analyze susceptibility data accurately, only data from hospitals that actively reported in specific years were included: 10 hospitals in 2016, 9 in 2017, 11 in 2018–2020, and 12 in 2021–2023. Contaminated and duplicate strains identified from the same patient were excluded from the analysis, when using WHONET 5.6 software for statistics.

### Strain cultivation and identification

Bacterial cultures and isolations followed standard microbiological diagnostic procedures. Isolates were identified using the VITEK automated bacterial analyzer (bioMérieux, Marcy l’Étoile, France), the MALDI-TOF/MS mass spectrometry identification instrument (Bruker, Champs-sur-Marne, France) or the BD Phoenix automated microbiology system (Becton Dickinson, Sparks, MD, USA). Identification tests were validated using control strains *E. coli* (ATCC 25922) and *S. aureus* (ATCC 29213).

### Antibiotic sensitivity testing

Antimicrobial susceptibility testing was performed using automated systems such as the VITEK2 Compact (France) and the BD Phoenix (Becton Dickinson) or the Kirby–Bauer disk diffusion method (Oxoid, Thermo Fisher, Pittsburg, PA, USA). The results were interpreted based on the Clinical and Laboratory Standards Institute (CLSI) M100-S33 guidelines (2023) (Clinical and Laboratory Standards Institute (CLSI),2023).The control strains for these procedures were *P. aeruginosa* (ATCC 27853), *E. coli* (ATCC 25922), *S. aureus* (ATCC 29213) and *Enterococcus faecalis* (ATCC 29212).

### Definitions

Neonatal patients: Defined as those ≤ 28 days old.
Pediatric patients: Defined as those aged 29 days to 14 years (Nichols et al., 2015).
COVID-19 period division: The study period (2016–2023) was divided into the pre-COVID-19 pandemic years (2016–2019) and post-COVID-19 pandemic years (2020–2023).
MRSA: Defined as resistant of oxacillin at a minimum inhibitory concentration (MIC) value of ≥ 4 μg/mL.
DTR (Difficult-to-Treat Resistance): A novel approach to defining resistance in Gram-negative bacteria that is focused on treatment-limiting resistance to first-line agents. The DTR phenotype for Gram-negative bacteria is an isolate that tests not susceptible (intermediate or resistant) to all β-lactam categories including carbapenems and fluoroquinolones and that isolates that were not susceptible to first-line agents were associated with increased patient mortality (Kadri et al., 2018).
ECR (Extended-spectrum cephalosporin resistance): Defined as resistance to cefotaxime, ceftazidime or cefepime *in vitro*.
CR (Carbapenem resistance): Defined as resistance to imipenem or meropenem *in vitro*.
AGR (Aminoglycoside resistance): Defined as resistance to gentamicin or amikacin *in vitro*.
FQR (Fluoroquinolone resistance): Defined as resistance to ciprofloxacin or levofloxacin *in vitro.*

### Statistical analysis

Antimicrobial resistance data were analyzed using WHONET 5.6 software to obtain the MIC_50_ and MIC_90_ values. Prism 8 (GraphPad, Boston, MA, USA) was used for statistical visualization. The temporal trends, age distributions, and detection levels of resistance phenotypes were analyzed using Fisher’s exact test or the Chi-square test. The two periods (before and after COVID-19) were compared use Chi-square test. To evaluate linear trends in detection rates and MIC values for the same drug across different years and groups, the Chi-square test for trend was applied. Dynamic changes in MIC values over time were assessed using the non-parametric Spearman correlation test. Statistical significance was established at a two-tailed P-value < 0.05. In the figures, statistically significant increases were represented in red color and decreases in a blue color.

## Results

### Changes in the distribution of ESKAPEEc BSIs

**Figure 1:**
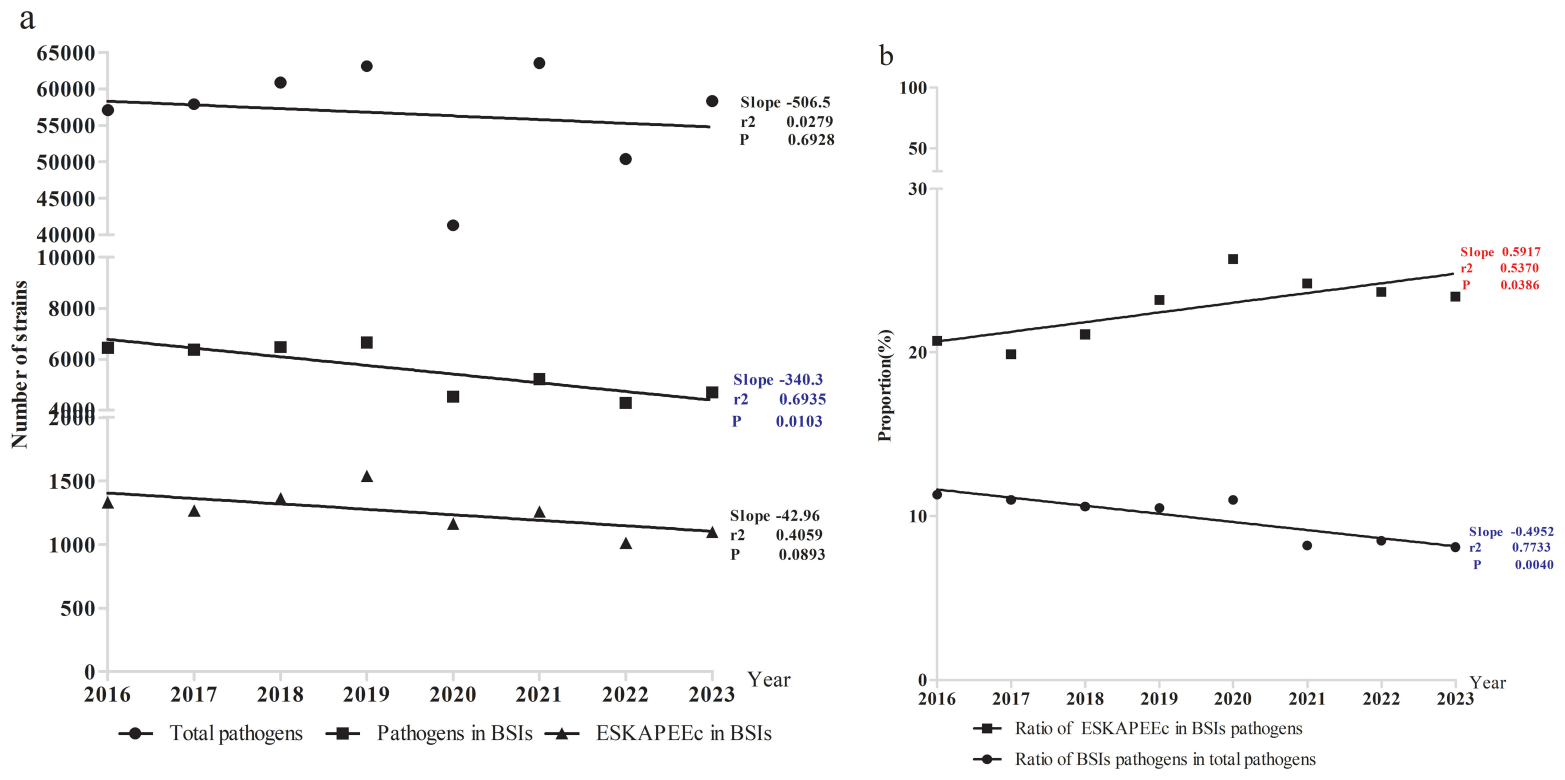
Incidence of pathogenic bacteria in bloodstream infections. **(a)** Pathogens number. **(b)** Percentage of BSIs and ESKAPEEc.

**Figure 2:**
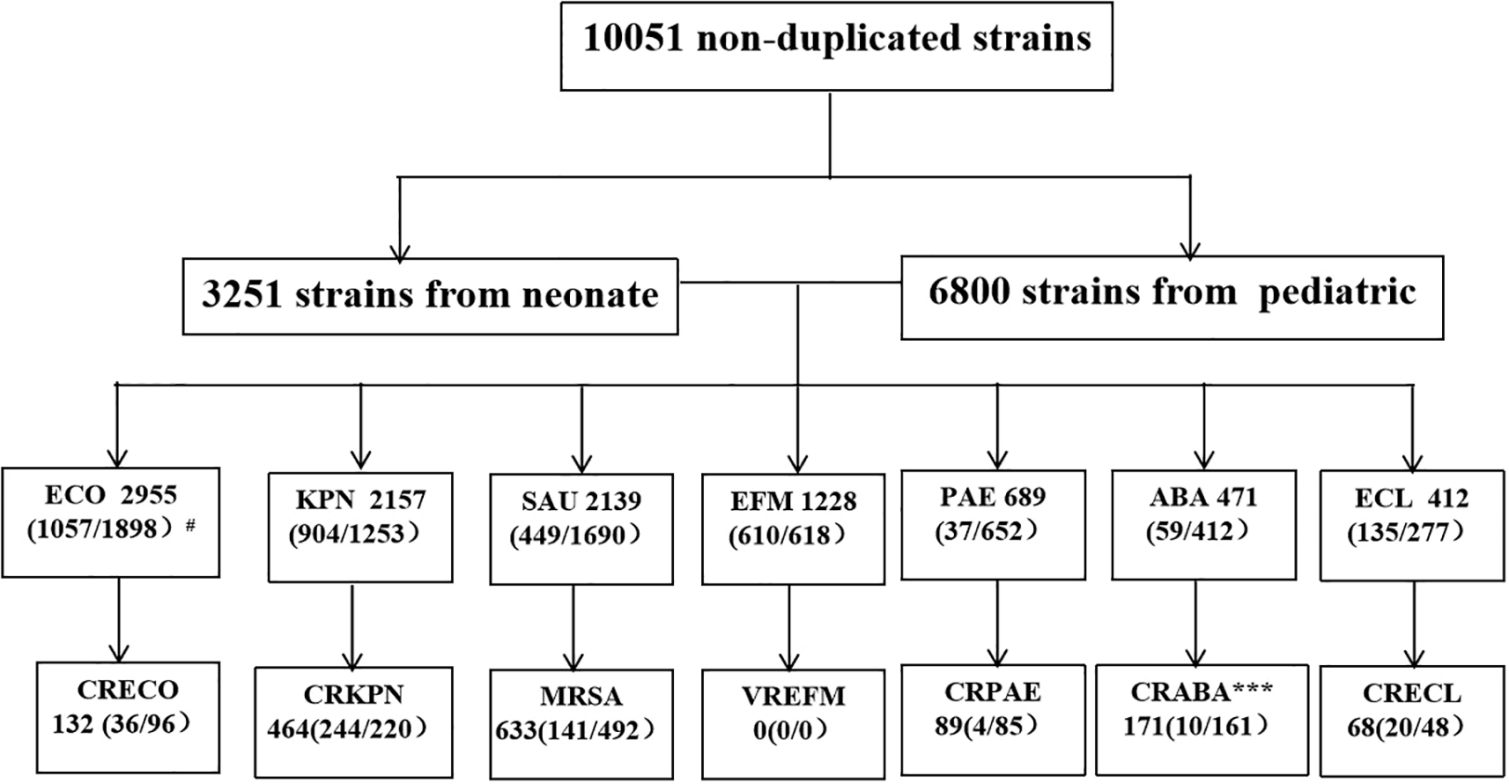
ESKAPEEc pathogens encountered in BSIs in this study. ECO, *E. coli*; KPN, *K. pneumoniae*; SAU, *S. aureus*; *EFM, E. faecium*; PAE, *P. aeruginosa*; ABA, *A. baumannii*; ECL, *Enterobacter cloacae(E. cloacae)*; CRECO, carbapenem-resistant *E. coli*; CRKPN, carbapenem-resistant *K. pneumoniae*; MRSA, methicillin-resistant *S. aureus*; VREFM, Vancomycin-resistant *E. faecium*; CRPAE, carbapenem-resistant *P. aeruginosa*; CRABA, carbapenem-resistant *A. baumannii*; CRECL, carbapenem-resistant *E. cloacae.* # N (A/B) where N indicates total number of strains in the BSIs and A the number of strains isolated from neonatal BSIs. B represents the number of strains isolated from Pediatric BSIs. * P < 0.05, *** P < 0.001.

This study identified a total of 452,435 pathogenic bacterial strains as part of the ISPED project. These included 44,675 bloodstream infection pathogens (9.9%) and the ESKAPEEc group accounted for 22.5% (10,051/44,675) of bloodstream infection pathogens ((**Figures 1A, B**). *Staphylococcus epidermidis* (26.9%) was the most frequently isolated bacteria, followed by *Staphylococcus hominis* (17.7%), *E. coli*(6.6%), *Stenotrophomonas maltophilia*(4.9%) and *K. pneumoniae*(4.8%).The incidence ranking of *S. aureus* and *Stenotrophomonas maltophilia* were different between ESKAPEEc pre-COVID-19 pandemic and post-COVID-19 pandemic, and neonates and pediatric cases (**Tables 1**, **2**). The incidence of CRABA had significantly differed in the pediatric and neonate groups (P < 0.05) (**Figure 2**).

**Table 1:** The distribution of the 20 most prevalent pathogens isolated from blood culture specimens from 2016–2019 and 2020-2023.

Pathogens	Overall			2016-2019			2020-2023		
	No of pathogens (n =44675)	Percentage(%)	Rank	No of pathogens (n = 25946)	Percentage(%)	Rank	No of pathogens (n =18729)	Percentage(%)	Rank
Gram-negative									
*E. coli*	2955	6.6	3	1563	6.0	3	1392	7.4	3
*Stenotrophomonas maltophilia*	2189	4.9	4	1434	5.5	4	755	4.0	6
*K.pneumoniae*	2157	4.8	5	1236	4.8	5	921	4.9	5
*Achromobacter xylosoxidans*	709	1.6	13	454	1.7	11	255	1.4	14
*P.aeruginosa*	684	1.5	14	361	1.4	13	328	1.8	13
*A.baumannii*	469	1.0	17	275	1.1	16	196	1.0	18
*E.cloacae*	409	0.9	18	207	0.8	18	205	1.1	17
*Salmonella* spp.	330	0.7	19	104	0.4	22	226	1.2	15
*Serratia marcescens*	296	0.7	20	189	0.7	19	107	0.6	20
Gram-positive									
*Staphylococcus epidermidis*	11998	26.9	1	7166	27.6	1	4845	25.9	1
*Staphylococcus hominis*	7919	17.7	2	4694	18.1	2	3232	17.3	2
*S.aureus*	2139	4.8	6	1098	4.2	6	1041	5.6	4
*Staphylococcus haemolyticus*	1614	3.6	7	1059	4.1	7	557	3.0	7
*E.faecium*	1228	2.7	8	771	3.0	8	457	2.4	10
*Staphylococcus capitis*	1129	2.5	9	599	2.3	10	530	2.8	8
*Streptococcus pneumoniae*	1072	2.4	10	687	2.6	9	385	2.1	12
*E.faecalis*	835	1.9	11	431	1.7	12	405	2.2	11
*Streptococcus mitis*	746	1.7	12	284	1.1	15	463	2.5	9
*Streptococcus agalactiae*	488	1.1	15	335	1.3	14	153	0.8	19
*Staphylococcus warneri*	482	1.1	16	260	1.0	17	222	1.2	16

**Table 2:** Age distribution of the 20 most prevalent pathogens from bloodstream infections reported from 2016 to 2023.

Pathogen	Overall			Neonatal			Pediatric		
	No of pathogens (n =44675)	Percentage(%)	Rank	No of pathogens (n = 12474)	Percentage(%)	Rank	No of pathogens (n =32201)	Percentage(%)	Rank
Gram-negative									
*E. coli*	2955	6.6	3	1057	8.5	2	1898	1.4	4
*Stenotrophomonas maltophilia*	2189	4.9	4	126	1.0	14	2063	1.6	3
*K.pneumoniae*	2157	4.8	5	904	7.2	4	1253	0.9	6
*Achromobacter xylosoxidans*	709	1.6	13	180	1.4	11	524	0.4	13
*P.aeruginosa*	684	1.5	14	37	0.3	21	652	0.5	11
*A.baumannii*	469	1.0	17	59	0.5	17	412	0.3	14
*E.cloacae*	409	0.9	18	135	1.1	13	277	0.2	18
*Salmonella* spp.	330	0.7	19	8	0.1	43	322	0.2	17
*Serratia marcescens*	296	0.7	20	112	0.9	15	184	0.1	19
Gram-positive									
*Staphylococcus epidermidis*	11998	26.9	1	4871	39.0	1	7104	5.4	1
*Staphylococcus hominis*	7919	17.7	2	1004	8.0	3	6911	5.2	2
*S.aureus*	2139	4.8	6	449	3.6	8	1690	1.3	5
*Staphylococcus haemolyticus*	1614	3.6	7	562	4.5	6	1051	0.8	8
*E.faecium*	1228	2.7	8	610	4.9	5	618	0.5	12
*Staphylococcus capitis*	1129	2.5	9	411	3.3	9	712	0.5	9
*Streptococcus pneumoniae*	1072	2.4	10	7	0.1	50	1065	0.8	7
*E.faecalis*	835	1.9	11	467	3.7	7	366	0.3	15
*Streptococcus mitis*	746	1.7	12	47	0.4	18	698	0.5	10
*Streptococcus agalactiae*	488	1.1	15	373	3.0	10	115	0.1	25
*Staphylococcus warneri*	482	1.1	16	140	1.1	12	341	0.3	16

**Figure 3:**
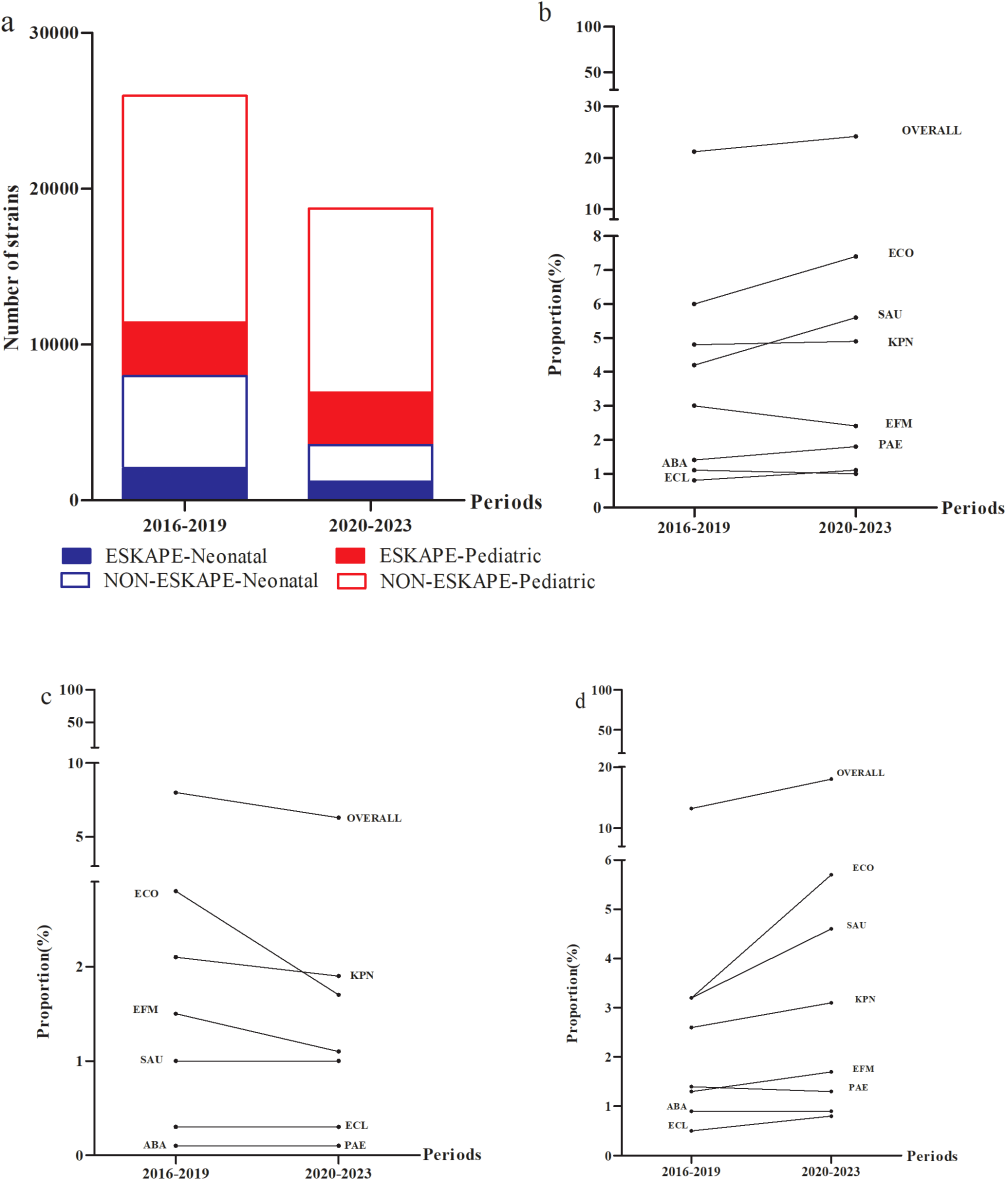
Temporal trends and age distributions of ESKAPEEc pathogens in bloodstream infections from 2016–2019 to 2020–2023. **(a)** General distribution of ESKAPEEc pathogens in BSIs. ESKAPEEc pathogens in **(b)** all BSIs **(c)** neonatal BSIs and **(d)** pediatric BSIs. ECO, *E*. *coli*; KPN, *K.pneumoniae*; ECL, *E.cloacae*; PAE, *P.aeruginosa*; ABA, *A*. *baumannii*; SAU, *S.aureus*; EFM, *E.faecium.*.

The number of ESKAPEEc pathogens fell from 5511 in 2016–2019 to 4540 in 2020-2023(**Figure 3A**). Notably, the average composition proportion of blood samples dropped from 10.4% in 2016–2019 to 8.7% in 2020–2023 (**Supplementary Table S1**). Interestingly, the proportion of ESKAPEEc significantly increased from 21.2% in 2016–2019 to 24.2% in 2020–2023 for total BSIs (**Figure 3B**). The proportion of ESKAPEEc decreased from 8.0% in 2016–2019 to 6.5% in 2020–2023 for neonatal BSIs (**Figure 3C**), although the proportion of ESKAPEEc increased from 13.2% in 2016–2019 to 18.0% in 2020–2023 for pediatric BSIs (**Figure 3D**).

### Trends in antimicrobial resistance patterns in ESKAPEEc over time

**Figure 4:**
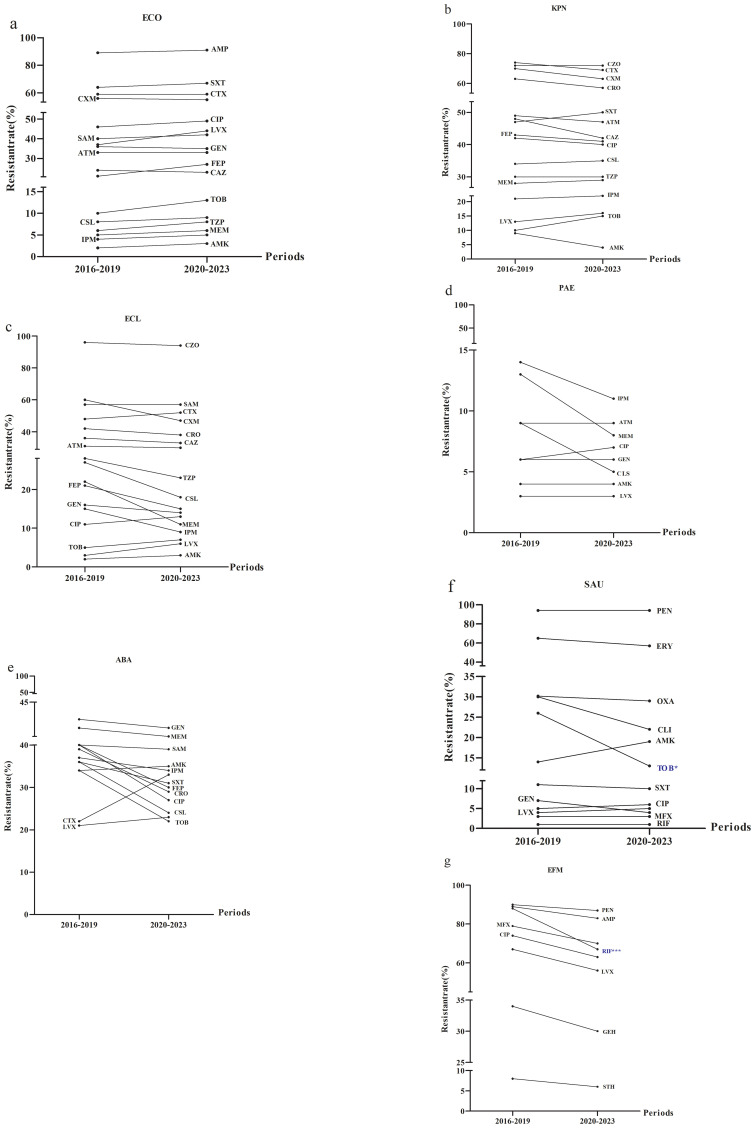
Alterations in antimicrobial resistance profiles of ESKAPEEc pathogens from 2016 to 2019 and 2020–2023. **(a)** ECO; **(b)** KPN; **(c)** ECL; **(d)** PAE; **(e)** ABA; **(f)** SAU; **(g)** EFM. ECO, *E*. *coli*; KPN, *K.pneumoniae*; ECL, *E.cloacae*; PAE, *P.aeruginosa*; ABA, *A*. *baumannii*; SAU, *S.aureus*; EFM, *E.faecium.*AMP, Ampicillin; CXM, cefuroxime; CTX, Cefotaxime; CAZ, Ceftazidime; FEP, Cefepime; SAM, Ampicillin/Sulbactam; TZP, Piperacillin/Tazobactam; CLS, Cefoperazone/Sulbactam; ATM, Aztreonam; IPM, Imipenem; MEM, Meropenem; CIP, Ciprofloxacin; LEV, Levofloxacin; AMK, Amikacin; GEN, Gentamicin; TOB, Tobramycin; SXT, Trimethoprim/Sulfamethoxazole; CZO, Cefazolin; CRO, Ceftriaxone; PEN, Penicillin G; OXA, Oxacillin; MXF, Moxifloxacin; ERY, Erythromycin; CLI, clindamycin; RIF, Rifampicin; STH, High-level streptomycin resistance; GEH, High-level gentamicin resistance. * P < 0.05, *** P < 0.001.

Given the observed increased in the incidence of ESKAPEEc over time, further investigation of AMR profiles was conducted. For *E. coli*, resistance to cefuroxime, ceftazidime and gentamicin decreased between the study periods of 2016–2019 and 2020-2023 (**Figure 4A**). *K. pneumoniae* exhibited increased resistance to tobramycin and trimethoprim/sulfamethoxazole during the same periods (**Figure 4B**). The resistance of *E. cloacae* to imipenem, meropenem, ceftazidime and cefepime was decreased (**Figure 4C**) while resistance of *P. aeruginosa* to imipenem and meropenem decreased (**Figure 4D**). Notably, *A. baumannii* displayed a reduction in resistance to most antibiotics tested whereas its resistance to cefotaxime, levofloxacin and amikacin increased (**Figure 4E**). For Gram-positive ESKAPEEc, *S. aureus* demonstrated rising resistance to ciprofloxacin and levofloxacin, whereas its resistance to penicillin G remained consistently high at 94.0% (**Figure 4F**). For *E. faecium*, resistance to all agents tested decreased, particularly resistance to rifampicin that dropped from 87.6% to 66.7% (P < 0.0001, **Figure 4G**).

### Antimicrobial resistance patterns of gram-negative ESKAPEEc by age

**Figure 5:**
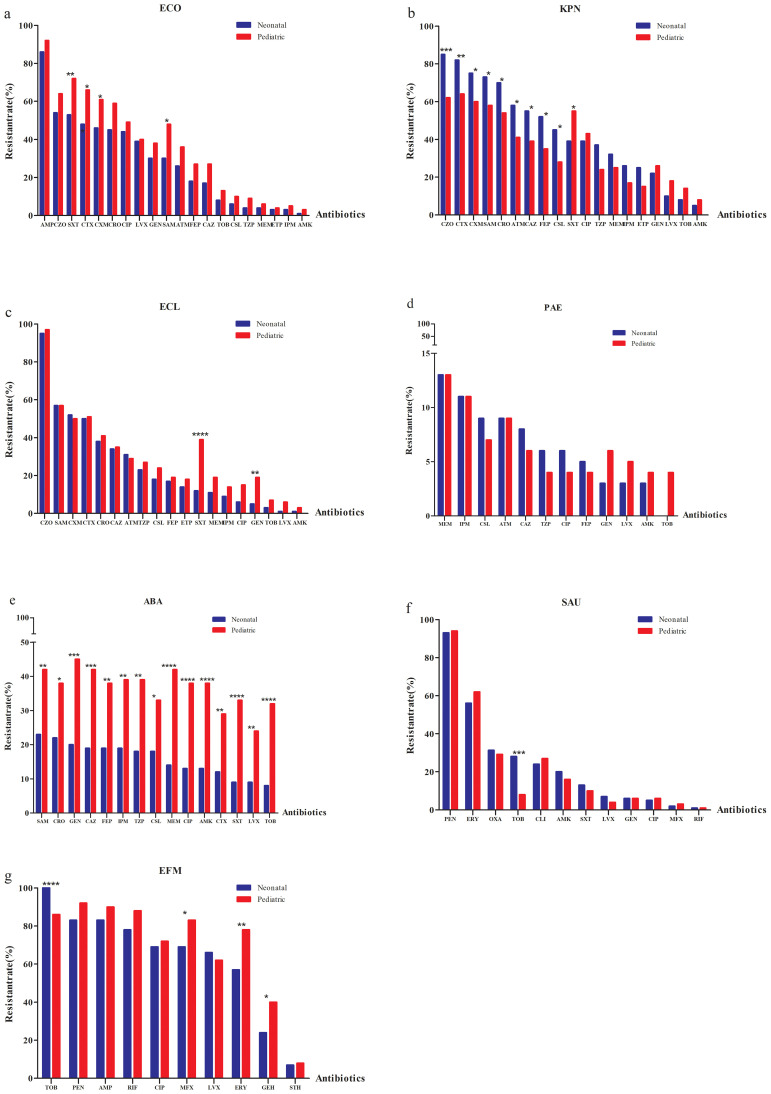
Antimicrobial resistance profiles of ESKAPEEc pathogens in neonatal and pediatric BSIs. **(a)** ECO; **(b)** KPN; **(c)** ECL; **(d)** PAE; **(e)** ABA; **(f)** SAU; **(g)** EFM. ECO, *E*. *coli*; KPN, *K.pneumoniae*; ECL, *E.cloacae*; PAE, *P.aeruginosa*; ABA, *A*. *baumannii*; SAU, *S.aureus*; EFM, *E.faecium;* AMP, Ampicillin;CXM, Cefuroxime; CTX, Cefotaxime; CAZ, Ceftazidime; FEP, Cefepime;SAM, Ampicillin/Sulbactam; TZP, Piperacillin/Tazobactam; CLS, Cefoperazone/Sulbactam; ATM, Aztreonam; IPM, Imipenem; MEM, Meropenem; CIP, Ciprofloxacin; LEV, Levofloxacin; AMK, Amikacin; GEN, Gentamicin;TOB,Tobramycin; SXT, Trimethoprim/Sulfamethoxazole; CZO, Cefazolin; CRO, Ceftriaxone; PEN, Penicillin G; OXA, Oxacillin; MXF, Moxifloxacin; ERY, Erythromycin; CLI, clindamycin; RIF, Rifampicin; STH, High-level streptomycin resistance; GEH, High-level gentamicin resistance. * P < 0.05, ** P < 0.01, *** P < 0.001.

Given the differences in ESKAPEEc distributions between neonatal and pediatric BSIs, we hypothesized that these pathogens would display distinct antibiograms for each group. For *E. coli*, resistance to all tested agents was more prevalent in pediatric BSIs than in neonatal BSIs. (**Figure 5A**). In contrast to *E. coli*, *K. pneumoniae* displayed different AMR profiles between these groups. Strains isolated from pediatric BSIs exhibited significantly lower resistance to all β-lactams compared to those from neonatal BSIs (**Figure 5B**). The resistance of *E. cloacae* to trimethoprim/sulfamethoxazole and gentamicin were significant higher in pediatric BSIs than in neonatal BSIs(P < 0.05) (**Figure 5C**).

The non-fermenting bacterial group included resistance of *P. Aeruginosa* to ceftazidime that was higher in neonatal (8.0%) versus pediatric BSIs (6.0%) (**Figure 5D**). *A. baumannii* isolated from pediatric BSIs demonstrated significantly higher resistance to all tested agents compared to those from neonatal BSIs (all P < 0.05, **Figure 5E**).

MRSA was more prevalent in neonatal BSIs (31.4%) compared to pediatric BSIs (29.1%). *S. aureus and E. faecium* isolated from neonatal BSIs exhibited significantly higher resistance to tobramycin than those from pediatric BSIs (P < 0.001) (**Figures 5F, G**). None of the *S. aureus* or *E. faecium* isolates from either group were resistant to vancomycin or linezolid.

### MIC drift of vancomycin and linezolid against *S. aureus*

According to their oxacillin MIC values (≥4), the *S. aureus* isolates were divided into MRSA and methicillin-susceptible *S. aureus* (MSSA) categories. To assess the 8-year trend in MIC values for drugs against *S. aureus* isolates, we categorized the isolates into two groups: MIC ≤1 µg/mL and MIC ≥ 2 µg/mL. A total of 2,139 *S. aureus* strains were isolated over the 8-year study period that were composed of 1,506 MSSA and 633 MRSA strains. MSSA strains possessing vancomycin MICs ≥ 2 μg/mL (6.1%,92/1506) was lower than that of MRSA strains (8.1%, 51/633). In addition, the annual change in *S. aureus* isolates exhibiting linezolid MIC values ≥2 μg/mL was not significantly different from MSSA strains (P > 0.05), but did significantly differ from MRSA strains (P<0.01)(**Table 3**).

**Table 3:** MIC distribution of vancomycin and linezolid in *S. aureus*.

Year	Strains	Vancomycin						Linezolid					
		MIC distribution (ug/mL)			Percentages of MIC ≥2	MIC_50_ (ug/mL)	MIC_90_ (ug/mL)	MIC distribution (ug/mL)			Percentages of MIC ≥2	MIC_50_ (ug/mL)	MIC_90_ (ug/mL)
		<=0.5	1	≥2				<=1	2	4			
2016													
*MRSA*	55	29	20	6	10.9	0.5	2	6	46	3	89.1	2	2
*MSSA*	181	106	67	8	4.4	0.5	1	18	154	9	90.1	2	2
2017													
*MRSA*	71	32	33	6	8.5	1	1	5	64	2	93.0	2	2
*MSSA*	182	92	85	5	2.7	1	1	23	155	4	87.4	2	2
2018													
*MRSA*	76	37	33	6	7.9	1	1	9	61	6	88.2	2	2
*MSSA*	181	108	64	9	5.0	0.5	1	22	152	7	87.8	2	2
2019													
*MRSA*	108	59	42	7	6.5	0.5	1	14	90	4	87.0	2	2
*MSSA*	244	139	85	20	8.2	0.5	1	27	208	9	88.9	2	2
2020													
*MRSA*	81	42	33	6	7.4	2	2	7	73	1	91.4	2	2
*MSSA*	177	83	81	13	7.3	1	1	13	157	7	92.7	2	2
2021													
*MRSA*	83	40	35	8	9.6	1	2	16	61	6	80.7	2	2
*MSSA*	202	74	112	16	7.9	1	1	25	166	11	87.6	2	2
2022													
*MRSA*	79	43	31	5	6.3	0.5	1	18	60	1	77.2	2	2
*MSSA*	151	78	67	6	4.0	0.5	1	26	123	2	82.8	2	2
2023													
*MRSA*	80	43	30	7	8.8	0.5	1	14	62	4	82.5	2	2
*MSSA*	188	86	87	15	8.0	1	1	23	153	12	87.8	2	2

### The distribution of special antimicrobial resistance phenotypes by time

**Figure 6:**
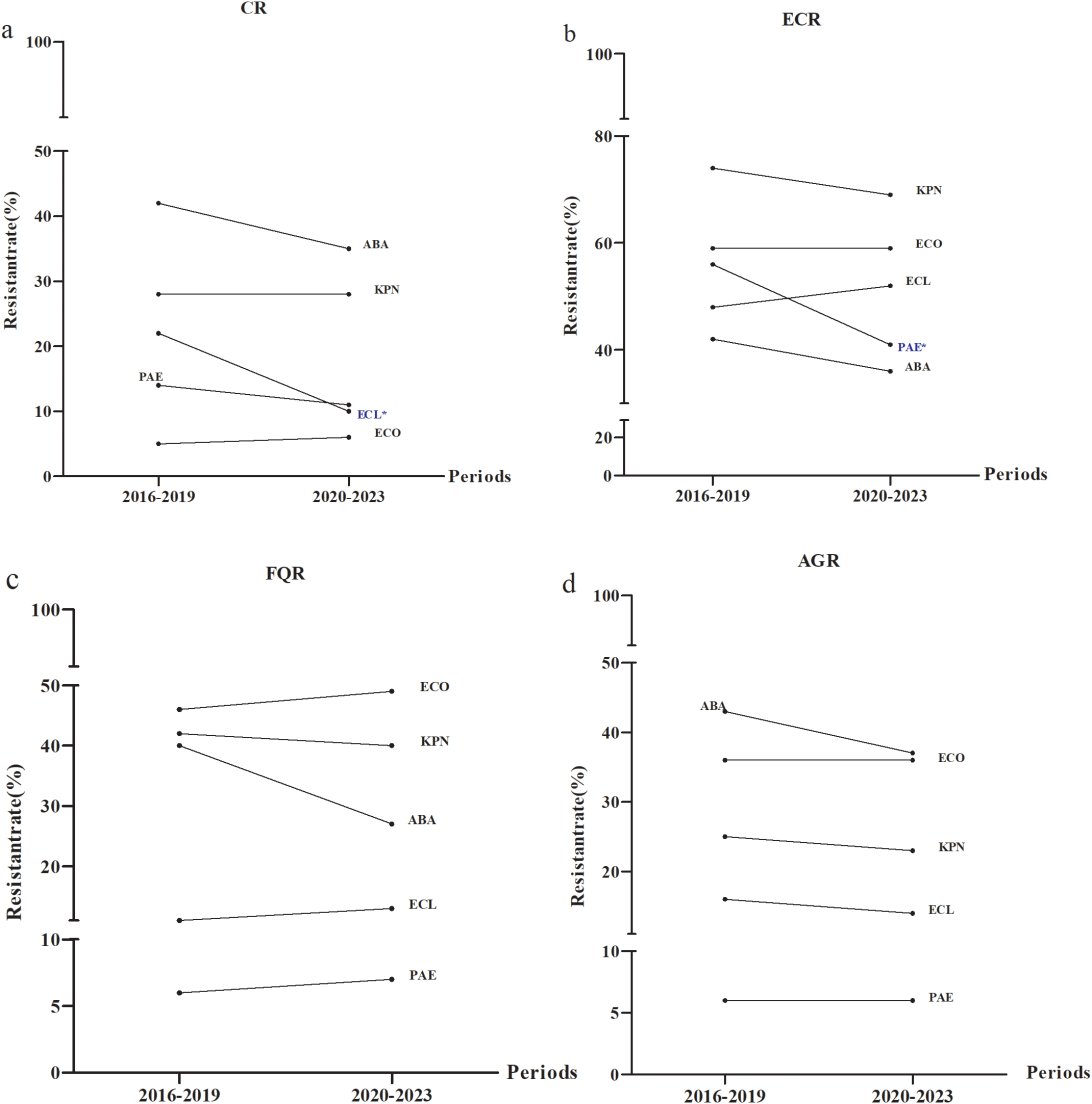
Distribution of special antimicrobial resistance phenotypes from 2016 to 2023. Temporal distributions of **(a)** CR **(b)** ECR **(c)** FQR and **(d)** AGR. ECO, *E*. *coli*; KPN, *K.pneumoniae*; ECL, *E.cloacae*; PAE, *P.aeruginosa*; ABA, *A*. *baumannii*; CR, carbapenem resistance; ECR, extended-spectrum cephalosporin resistance; FQR, fluoroquinolone resistance; AGR, aminoglycosides resistance. * P < 0.05.

The DTR detection levels for *E. coli, K. pneumoniae, E. cloacae, P. aeruginosa* and *A. baumannii* were 0% between 2016 and 2023. CR detection for *E. cloacae* manifested a downward trend from 2016–2019 to 2020-2023 (P< 0.05) (**Figure 6A**).The highest frequency of ECR and FQR were found for *K. pneumoniae* and *E. coli*, respectively (**Figures 6B, C**). AGR incidence was greatest for *A. baumannii* and the lowest for *P. Aeruginosa* (**Figure 6D**).

## Discussion

This study examined the temporal shifts in the distribution of ESKAPEEc pathogens and AMR patterns in neonatal and pediatric BSIs across China. Our multicenter study confirmed that the incidence of ESKAPEEc in BSIs was totally increased. along with shifts in AMR patterns, changes in vancomycin and linezolid MICs for *S. aureus* and variations in resistance patterns to four specific drugs in pediatric BSIs across China. As far as we know, this is the first multicenter study on ESKAPEEc in pediatric BSIs conducted in China, offering essential insights for optimizing antibiotic treatment strategies in pediatric BSIs.

The incidence of ESKAPEEc as a causative agent in BSIs was 22.5% from 2016 to 2023 and this was lower than the 65.3% reported for adult BSIs in southwest China (Yang et al., 2019) and the 61.7% adult BSIs in Rome (De Angelis et al., 2018). However, our findings align with an 11-year retrospective study on pediatric BSIs in the United States (Larru et al., 2016) that reported ESKAPEEc pathogens were responsible for 27.2% of all identified infections. This suggested that the distribution of pathogens in pediatric BSIs differs from that in adults and further emphasizes the need for further understanding of the pathogens involved in pediatric BSIs. Blood culture specimens submitted for examination decreased proportionally from 2016–2020 to 2020-2023 (10.4% vs. 8.7%) and the pathogen numbers for the BSIs decreased from 25946 to 18729 and ESKAPEEc pathogen numbers for BSIs also declined (5511 vs. 4540) even though the proportion of ESKAPEEc pathogens increased. The primary reason for this was significant increases in the proportions of *E. coli*, *P. aeruginosa* and *S. aureus* in 2020-2023.

*E. coli* was among the five most prevalent Gram-negative bacteria isolated in both neonatal and pediatric BSIs and was consistent with previous domestic and international studies (Crichton et al., 2018; Lyu et al., 2023; Sajedi Moghaddam et al., 2024; Wen et al., 2024; Yilmaz et al., 2025). The five most prevalent overall were *Staphylococcus epidermidis, Staphylococcus hominis*, *E. coli*, *Stenotrophomonas maltophilia* and *K. pneumoniae* and differed from a study conducted in Beijing, China that ranked the incidence as *K. pneumoniae*, *S. aureus*, *E. coli*, *E. faecium* and coagulase-negative *Staphylococcus* (Lyu et al., 2023). In our neonatal group, the five most prevalent were *Staphylococcus epidermidis*, *E. coli*, *Staphylococcus hominis*, *K. pneumoniae* and *E. faecium.* These results were consistent with a previous study (Wu et al., 2025) and that coagulase-negative *Staphylococcus* was the primary causative agent of neonatal sepsis in China and to a lesser extent *Enterobacter* and *Klebsiella* (Yang et al., 2024; Yu et al., 2022).We hypothesize that age and regional differences in climate, economy and health care conditions may explain the variation in BSIs pathogens globally. In support of this, COVID-19 contributed to around 1/6^th^ of sepsis-associated deaths in the United States between 2020 and 2022 and accounted for most of the excess sepsis associated mortality during the pandemic (Morrissey et al., 2025). COVID-19 also has affected BSI incidence in Chinese children. We observed the overall number of BSIs cases significantly declined post-COVID with a significant reduction in the constituent proportion of blood samples (10.4 versus 8.7%). A comparable reduction was also seen in the primary bacteria isolated from blood samples that included *E. coli* and *K. pneumoniae.* Similar findings were reported in Malawi where a notable reduction in Enterobacteriaceae incidence in BSIs was observed (Musicha et al., 2017). However, these results differ from a nine-year study in Rome (De Angelis et al., 2018). This discrepancy may be partially due to the heterogeneity in study design since our study was conducted in 12 tertiary children’s hospitals across nine provinces and autonomous cities in China and provided a more extensive geographical coverage. Our also study attempted to analyze the changes in ESKAPEEc detection before and after the COVID-19 pandemic. The results demonstrated statistically significant differences and suggested that pandemic control measures such as hand hygiene may have played a role in reducing ESKAPEEc infections.

Carbapenem resistance for our study in *E. coli* was 5.5% and was similar to the 6.0% reported in the Jiangxi region of China (Zhou et al., 2023) while lower than the 12.9% reported in Beijing (Lyu et al., 2023) but higher than the 2.0% reported in pediatric BSIs in southwest China (Yang et al., 2019). It is noteworthy that carbapenem resistance of *E. coli* increased post-COVID and higher resistance levels were observed in the pediatric versus neonatal BSI groups. Moreover, resistance to imipenem, meropenem, cefoperazone/sulbactam and piperacillin/tazobactam were all <10.0% indicating that these antibiotics remained effective for the treatment of pediatric BSIs in our study.

Carbapenem resistance of *K. pneumoniae* in this study was 28.0% and was lower than the 50.8% previously reported (Lyu et al., 2023) while higher than the 13.4 - 23% among Chinese children (Fu et al., 2021). This variation could be due to the diversity of resistance elements between strains and the spread of conserved mobile genetic elements. Similar to *E. coli*, carbapenem resistance of *K. pneumoniae* increased post-COVID-19. We found that the antibiotic usage has changed pre- and post-COVID in China. Ceftazidime-avibactam and aztreonam-avibactam was used more frequently post-COVID (Ran et al., 2025). In particular, detection of CRKPN in the pediatric group (17.6%) was lower than that in the neonate group (27.0%) and may be associated with the weakened immune defenses in neonates as well as the use of central venous catheters, indwelling urinary catheters and prolonged administration of broad-spectrum antibiotics. These are all factors recognized to heighten the risk of carbapenem-resistant Enterobacteriaceae (Moghnieh et al., 2021). The average resistance levels of *K. pneumoniae* to ceftriaxone, ceftazidime and cefepime were 59.9%, 45.2% and 41.9% from 2016 to 2023, respectively. These were significantly lower than the 77.3%, 70.2% and 72.8% reported in Beijing Children’s Hospital (Lyu et al., 2023). Compared to 2016-2019, the resistance of *K. pneumoniae* to imipenem and meropenem increased in the period 2020-2023, while resistance to third- and fourth-generation cephalosporins decreased similar to previous findings (Tian et al., 2018). One possible explanation for this shift is that during severe infections, clinicians often prefer imipenem or meropenem over third- or fourth-generation cephalosporins, especially in younger patients, which leads to selective antibiotic pressure. The increased use of carbapenems stimulates carbapenemase production, which in turn contributes to the rise in CRKPN cases (Meyer et al., 2010; Yang et al., 2018; Zhang et al., 2019). We hypothesize that this may explain the rapid rise in CRKPN detection with higher levels in children compared to adults and higher in neonates compared to children.

Compared to 2016-2019, resistance of *E. cloacae* to meropenem significantly decreased in 2020-2023.The value was lower than the 38.5% for meropenem previously reported (Lyu et al., 2023). Resistance for all tested antibiotics were higher in the pediatric group compared to the neonate group. In our study, *E. cloacae* displayed higher resistance incidence to piperacillin/tazobactam, cefoperazone/sulbactam, imipenem, meropenem and ceftazidime compared to *E. coli*, but lower than *K. pneumoniae*. As a result, clinicians should choose the most suitable antibiotics based on the identification of the bacteria and the results of antibiotic susceptibility testing.

*P. aeruginosa* resistance to imipenem, meropenem or aztreonam was <15.0%, which is consistent with our previous research (Xu et al., 2024) while lower than children in the Beijing, China report (Lyu et al., 2023), These data suggested that these antibiotics can be considered as first-line treatment for pediatric BSIs caused by *P. aeruginosa*. Additionally, resistance of *A. baumannii* to ciprofloxacin and amikacin were >25.0% and this exceeded the levels in an Australian children study (Williams et al., 2024).As the proportion of *A. baumannii* in BSIs declined, resistance to most antibiotics also decreased, particularly the resistance to imipenem, which fell from 37.0 to 33.5% and was consistent with the 37.8% reported previously in Beijing (Lyu et al., 2023). The decline in carbapenem resistance may be associated with the implementation of strategies designed to combat carbapenem-resistant organisms that included environmental monitoring, colonization clearance and resistance surveillance. Despite the overall decline, resistance of *A. baumannii* for all antibiotics tested were notably higher in the pediatric group compared to the neonate group (P<0.05). This was particularly notable for imipenem and meropenem resistance that were 39.2 and 41.6% for pediatric BSIs and 18.9 and 14.3% for neonate BSIs, respectively. Consequently, it is crucial to develop effective treatment strategies to tackle BSIs caused by *A. baumannii*.

In our study, the proportion of MRSA was 29.6% and was higher than the 15% reported in the Australian children study (Williams et al., 2025) but lower than the 34.0% reported in Zhejiang, China (Zheng et al., 2023) and 34.0% in Iran (Sajedi Moghaddam et al., 2024), but consistent with the 30.0% reported in Beijing (Lyu et al., 2023). MRSA detection in the neonate group was 31.4% versus 29.1% in the pediatric group, but significantly lower than the 85% reported in a tertiary neonatal unit in South Africa (Thomas et al., 2024). Effective antimicrobial resistance monitoring is crucial in the treatment of MRSA infections and strict measures should be enforced to prevent the transmission of MRSA.

Vancomycin is a glycopeptide antibiotic and a member of a group of glycosylated cyclic or polycyclic non-ribosomal peptides that inhibit cell wall synthesis in Gram-positive bacteria (Zeng et al., 2016). Several studies have observed a gradual increase in the MIC of vancomycin against *S. aureus* designated as the vancomycin ‘MIC creep’. However, this phenomenon remains controversial due to inconsistent and inconclusive findings (Jian et al., 2020). A previous study investigated the correlation between glycopeptide MICs and clinical outcomes of *S. aureus* infections and suggested that changes in glycopeptide MIC values should be considered when treating MRSA infections, particularly in patients who fail to achieve relief after glycopeptide treatment (Chen et al., 2013). Our findings indicated that the vancomycin MICs in MRSA strains remained stable over time in contrast to another report (Haas et al., 2023). Additionally, while both the MIC_50_ and MIC_90_ values remained relatively stable, the proportion of MSSA isolates with a vancomycin MIC ≥2 μg/mL increased over time. This contrasts with findings from other studies that observed a shift in vancomycin MICs against MSSA (Haas et al., 2023; Jian et al., 2020). The yearly change in *S. aureus* isolates exhibiting linezolid MIC values ≥2 μg/mL was not significantly different in MSSA strains, but it was significantly different in MRSA strains. However, the MIC_50_ and MIC_90_ values of both MRSA and MSSA remained unchanged and linezolid resistance in MRSA and MSSA displayed no statistically significant trends of ‘MIC creep’. All *E. faecium* strains were sensitive to vancomycin and were consistent with the susceptibility patterns in a domestic study (Wang et al., 2021) but higher than those reported in an overseas study (Sajedi Moghaddam et al., 2024). Sensitivity to high-level streptomycin resistance was >90% versus 88% reported previously (Sajedi Moghaddam et al., 2024). In our study, we observed that the proportion of MRSA strains with a vancomycin MIC of ≥2 μg/mL was higher than that of MSSA strains, while the proportion of MRSA strains with a linezolid MIC of ≥2 μg/mL was lower compared to MSSA strains. This finding warrants further investigation.

The emergence of DTR has presented a significant challenge to clinical infection control and hospital prevention efforts, making it a key focus of monitoring within public health services (Tamma et al., 2022). Validation in large patient cohorts has demonstrated that this new definition may more accurately reflect its association with clinical outcomes and aid in the design and assessment of clinical trials for managing drug-resistant Gram-negative infections (Tamma et al., 2022). This study did not detect any DTR strains and differed from the multicenter surveillance report in Hubei province (Zhang et al., 2022). Detection of CRABA and CRPAE were 38.5% and 12.5%, respectively and were lower than the 54.5 and 26.9% reported in the Beijing Children’s Hospital study (Lyu et al., 2023). Detection rates for CRABA and CRPAE in 2016–2019 were higher than those observed in 2020–2023 while the trend for CRECO displayed the opposite pattern. ECR detection indicated *K. pneumoniae* with the highest incidence followed by *E. coli*. Compared to 2016-2019, the detection of ECR-KPN, ECR-ABA and ECR-PAE displayed a decreasing trend while ECR-ECO and ECR-ECL levels increased in 2020-2023. The detection of FQR-ECO and AGR-ECO were significantly higher than those of FQR-KPN and AGR-KPN, and those for FQR-ABA and AGR-ABA were notably higher than FQR-PAE and AGR-PAE respectively. These were all lower than the Beijing study (Lyu et al., 2023). The low resistance to fluoroquinolones and aminoglycosides was related to their limited clinical use in children. Fluoroquinolones can cause significant side effects on children’s bones, potentially damaging joints, while aminoglycosides have ototoxic effects, which may cause hearing impairment. As a result, these drugs are rarely used in children. However, when clear clinical indications exist and no alternative antibiotics with lower toxicity are available, these drugs may be considered. However, their indications, dosage and treatment course should be strictly controlled and adverse reactions should be closely monitored to ensure the safety of the treatment.

## Conclusions

The distribution and AMR profiles of ESKAPEEc pathogens have evolved over time and show differences between neonatal and pediatric BSIs. *E. coli* is the most predominant pathogen in pediatric BSIs within ESKAPEEc followed by *K. pneumoniae* and *S. aureus*. There was an increased in the incidence of ESKAPEEc from 2016–2019 to 2020-2023. For Gram-negative ESKAPEEc, compared to 2016-2019, resistance to carbapenems decreased for *E. cloacae*, *A. baumannii* and *P. aeruginosa* from 2020–2023 while *E. coli* showed an increasing trend. With the exception of *K. pneumoniae* that exhibited higher carbapenem resistance levels in the neonatal group compared to the pediatric group, carbapenem resistance was higher in the pediatric group than in the neonatal group for the other ESKAPEEc pathogens. This suggested that the AMR patterns of ESKAPEEc are age-dependent and carbapenem drugs remain effective in the treatment of Gram-negative ESKAPEEc BSIs. All Gram-positive ESKAPEEc strains were sensitive to vancomycin and linezolid. The MICs for glycopeptides in MRSA strains remained stable over time, whereas MSSA strains showed a shift in vancomycin MICs. Linezolid showed no statistically distinct trends of ‘MIC creep’ in both MRSA and MSSA strains. The most frequently detected bacteria in CR, ECR, FQR and AGR were *A. baumannii*, *K. pneumoniae*, *E. coli* and *A. baumannii*, respectively.

## Limitation

This study has a little of limitation. Changes in diagnostic practices, blood culture submission rates, and antibiotic usage policies during COVID-19 could have potential influence.

## Data Availability

The datasets presented in this study can be found in online repositories. The names of the repository/repositories and accession number(s) can be found in the article/**Supplementary Material**.

## References

[B1] Buetti N. Marschall J. Atkinson A. Kronenberg A. (2016). National bloodstream infection surveillance in Switzerland 2008-2014: different patterns and trends for university and community hospitals. Infection control Hosp. Epidemiol. 37, 1060–1067. doi: 10.1017/ice.2016.137, PMID: 27350313

[B2] Chen K. Chang H. Hsu P. Yang C. Chia J. Wu T.. (2013). Relationship of teicoplanin MICs to treatment failure in teicoplanin-treated patients with methicillin-resistant Staphylococcus aureus pneumonia. J. microbiology immunology infection = Wei mian yu gan ran za zhi 46, 210–216. doi: 10.1016/j.jmii.2012.06.010, PMID: 22999099

[B3] Clinical and Laboratory Standards Institute (CLSI) (2023). Performance standards for antimicrobial susceptibility testing. 33th (Wayne, PA: CLSI). CLSI supplement M100.

[B4] Crichton H. O’Connell N. Rabie H. Whitelaw A. Dramowski A. (2018). Neonatal and paediatric bloodstream infections: Pathogens, antimicrobial resistance patterns and prescribing practice at Khayelitsha District Hospital, Cape Town, South Africa. South Afr. Med. J. = Suid-Afrikaanse tydskrif vir geneeskunde 108, 99–104. doi: 10.7196/SAMJ.2017.v108i2.12601, PMID: 29429440

[B5] De Angelis G. Fiori B. Menchinelli G. D’Inzeo T. Liotti F. Morandotti G.. (2018). Incidence and antimicrobial resistance trends in bloodstream infections caused by ESKAPE and Escherichia coli at a large teaching hospital in Rome, a 9-year analysis, (2007-2015). Eur. J. Clin. Microbiol. Infect. Dis. 37, 1627–1636. doi: 10.1007/s10096-018-3292-9, PMID: 29948360

[B6] Folgori L. Livadiotti S. Carletti M. Bielicki J. Pontrelli G. Ciofi Degli Atti M.. (2014). Epidemiology and clinical outcomes of multidrug-resistant, gram-negative bloodstream infections in a European tertiary pediatric hospital during a 12-month period. Pediatr. Infect. Dis. J. 33, 929–932. doi: 10.1097/inf.0000000000000339, PMID: 24642515

[B7] Founou R. Founou L. Essack S. (2017). Clinical and economic impact of antibiotic resistance in developing countries: A systematic review and meta-analysis. PloS One 12, e0189621. doi: 10.1371/journal.pone.0189621, PMID: 29267306 PMC5739407

[B8] Fu P. Xu H. Jing C. Deng J. Wang H. Hua C.. (2021). Bacterial epidemiology and antimicrobial resistance profiles in children reported by the ISPED program in China 2016 to 2020. Microbiol. Spectr. 9, e0028321. doi: 10.1128/Spectrum.00283-21, PMID: 34730410 PMC8567242

[B9] Gupta M. Gupta V. Gupta R. Chaudhary J. (2024). Current trends in antimicrobial resistance of ESKAPEEc pathogens from bloodstream infections - Experience of a tertiary care centre in North India. Indian J. Med. Microbiol. 50, 100647. doi: 10.1016/j.ijmmb.2024.100647, PMID: 38871082

[B10] Haas K. Meyer-Buehn M. von Both U. Hübner J. Schober T. (2023). Decrease in vancomycin MICs and prevalence of hGISA in MRSA and MSSA isolates from a German pediatric tertiary care center. Infection 51, 583–588. doi: 10.1007/s15010-023-02036-5, PMID: 37072604 PMC10205833

[B11] Hu F. Guo Y. Zhu D. Wang F. Jiang X. Xu Y.. (2016). Resistance trends among clinical isolates in China reported from CHINET surveillance of bacterial resistance 2005-2014. Clin. Microbiol. Infect. 22 Suppl 1, S9–14. doi: 10.1016/j.cmi.2016.01.001, PMID: 27000156

[B12] Jian Y. Lv H. Liu J. Huang Q. Liu Y. Liu Q.. (2020). Staphylococcus aureusDynamic changes of susceptibility to vancomycin, teicoplanin, and linezolid in a central teaching hospital in Shanghai, China 2008-2018. Front. Microbiol. 11. doi: 10.3389/fmicb.2020.00908, PMID: 32528428 PMC7247803

[B13] Kadri S. Adjemian J. Lai Y. Spaulding A. Ricotta E. Prevots D.. (2018). Difficult-to-treat resistance in gram-negative bacteremia at 173 US hospitals: retrospective cohort analysis of prevalence, predictors, and outcome of resistance to all first-line agents. Clin. Infect. Dis. 67, 1803–1814. doi: 10.1093/cid/ciy378, PMID: 30052813 PMC6260171

[B14] Kargaltseva N. Borisova O. Mironov A. Kocherovets V. Pimenova A. Gadua N. (2022). Bloodstream infection in hospital therapeutic patients. Klinicheskaia laboratornaia diagnostika 67, 355–361. doi: 10.51620/0869-2084-2022-67-6-355-361, PMID: 35749601

[B15] Kern W. Rieg S. (2020). Burden of bacterial bloodstream infection-a brief update on epidemiology and significance of multidrug-resistant pathogens. Clin. Microbiol. infection 26, 151–157. doi: 10.1016/j.cmi.2019.10.031, PMID: 31712069

[B16] Kontula K. Skogberg K. Ollgren J. Järvinen A. Lyytikäinen O. (2018). The outcome and timing of death of 17,767 nosocomial bloodstream infections in acute care hospitals in Finland during 1999-2014. Eur. J. Clin. Microbiol. Infect. Dis. 37, 945–952. doi: 10.1007/s10096-018-3211-0, PMID: 29455272

[B17] Kritsotakis E. Lagoutari D. Michailellis E. Georgakakis I. Gikas A. (2022). Burden of multidrug and extensively drug-resistant ESKAPEE pathogens in a secondary hospital care setting in Greece. Epidemiol. infection 150, e170. doi: 10.1017/s0950268822001492, PMID: 36148865 PMC9981128

[B18] Larru B. Gong W. Vendetti N. Sullivan K. Localio R. Zaoutis T.. (2016). Bloodstream infections in hospitalized children: epidemiology and antimicrobial susceptibilities. Pediatr. Infect. Dis. J. 35, 507–510. doi: 10.1097/inf.0000000000001057, PMID: 26766146

[B19] Lindell R. Nishisaki A. Weiss S. Traynor D. Fitzgerald J. (2020). Risk of mortality in immunocompromised children with severe sepsis and septic shock. Crit. Care Med. 48, 1026–1033. doi: 10.1097/ccm.0000000000004329, PMID: 32301846 PMC7311286

[B20] Lyu Z. Zhen J. Meng Q. Zhou W. An J. Dong F. (2023). Bacterial etiology and antimicrobial resistance pattern of pediatric bloodstream infections in Beijing 2015-2019. Infection Drug resistance 16, 6297–6308. doi: 10.2147/idr.S426000, PMID: 37780532 PMC10540788

[B21] Marturano J. Lowery T. (2019). ESKAPE pathogens in bloodstream infections are associated with higher cost and mortality but can be predicted using diagnoses upon admission. Open Forum Infect. Dis. 6, ofz503. doi: 10.1093/ofid/ofz503, PMID: 31844639 PMC6902016

[B22] Meyer E. Schwab F. Schroeren-Boersch B. Gastmeier P. (2010). Dramatic increase of third-generation cephalosporin-resistant E. coli in German intensive care units: secular trends in antibiotic drug use and bacterial resistance 2001 to 2008. Crit. Care (London England) 14, R113. doi: 10.1186/cc9062, PMID: 20546564 PMC2911759

[B23] Miller W. Arias C. (2024). ESKAPE pathogens: antimicrobial resistance, epidemiology, clinical impact and therapeutics. Nat. Rev. Microbiol. 22, 598–616. doi: 10.1038/s41579-024-01054-w, PMID: 38831030 PMC13147291

[B24] Moghnieh R. Abdallah D. Jadayel M. Zorkot W. El Masri H. Dib M.. (2021). Epidemiology, risk factors, and prediction score of carbapenem resistance among inpatients colonized or infected with 3rd generation cephalosporin resistant Enterobacterales. Sci. Rep. 11, 14757. doi: 10.1038/s41598-021-94295-1, PMID: 34285312 PMC8292374

[B25] Morrissey R. Lee J. Baral N. Tauseef A. Sood A. Mirza M.. (2025). Demographic and regional trends of sepsis mortality in the United States 1999-2022. BMC Infect. Dis. 25, 504. doi: 10.1186/s12879-025-10921-7, PMID: 40211200 PMC11987265

[B26] Musicha P. Cornick J. Bar-Zeev N. French N. Masesa C. Denis B.. (2017). Trends in antimicrobial resistance in bloodstream infection isolates at a large urban hospital in Malawi, (1998-2016): a surveillance study. Lancet Infect. Dis. 17, 1042–1052. doi: 10.1016/s1473-3099(17)30394-8, PMID: 28818544 PMC5610140

[B27] Nichols C. Cruz Espinoza L. von Kalckreuth V. Aaby P. Ahmed El Tayeb M. Ali M.. (2015). Bloodstream infections and frequency of pretreatment associated with age and hospitalization status in Sub-Saharan Africa. Clin. Infect. Dis. 61 Suppl 4, S372–S379. doi: 10.1093/cid/civ730, PMID: 26449954 PMC4596935

[B28] Niederman M. Baron R. Bouadma L. Calandra T. Daneman N. DeWaele J.. (2021). Initial antimicrobial management of sepsis. Crit. Care (London England) 25, 307. doi: 10.1186/s13054-021-03736-w, PMID: 34446092 PMC8390082

[B29] Péan de Ponfilly G. Benmansour H. Manda V. Lecorche E. Mougari F. Munier A.. (2021). Impact of 24/7 loading of blood culture bottles in a new automated incubator on the diagnosis of bloodstream infections. Eur. J. Clin. Microbiol. Infect. Dis. 40, 2639–2643. doi: 10.1007/s10096-021-04283-6, PMID: 34059934

[B30] Pogue J. Kaye K. Cohen D. Marchaim D. (2015). Appropriate antimicrobial therapy in the era of multidrug-resistant human pathogens. Clin. Microbiol. infection 21, 302–312. doi: 10.1016/j.cmi.2014.12.025, PMID: 25743999

[B31] Qin X. Ding L. Hao M. Li P. Hu F. Wang M. (2024). Antimicrobial resistance of clinical bacterial isolates in China: current status and trends. JAC-antimicrobial resistance 6, dlae052. doi: 10.1093/jacamr/dlae052, PMID: 38549710 PMC10977948

[B32] Ran X. Chen X. Wang C. Wang H. Xie W. Jing C. (2025). Klebsiella pneumoniaeCarbapenem-resistant infections in Chinese children: activities of ceftazidime-avibactam and aztreonam-avibactam against carbapenemase-producing strains in a two-center study. Front. Cell. infection Microbiol. 15. doi: 10.3389/fcimb.2025.1545999, PMID: 40207055 PMC11979245

[B33] Sajedi Moghaddam S. Mamishi S. Pourakbari B. Mahmoudi S. (2024). Bacterial etiology and antimicrobial resistance pattern of pediatric bloodstream infections: a 5-year experience in an Iranian referral hospital. BMC Infect. Dis. 24, 373. doi: 10.1186/s12879-024-09260-w, PMID: 38565980 PMC10988941

[B34] Tamma P. Aitken S. Bonomo R. Mathers A. van Duin D. Clancy C. (2022). Infectious Diseases Society of America 2022 Guidance on the Treatment of Extended-Spectrum β-lactamase Producing Enterobacterales (ESBL-E), Carbapenem-Resistant Enterobacterales (CRE), and Pseudomonas aeruginosa with Difficult-to-Treat Resistance (DTR-P. aeruginosa). Clin. Infect. Dis. 75, 187–212. doi: 10.1093/cid/ciac268, PMID: 35439291 PMC9890506

[B35] Thomas R. Ondongo-Ezhet C. Motsoaledi N. Sharland M. Clements M. Velaphi S. (2024). Incidence, pathogens and antimicrobial resistance of blood and cerebrospinal fluid isolates from a tertiary neonatal unit in South Africa: A 10 year retrospective review. PloS One 19, e0297371. doi: 10.1371/journal.pone.0297371, PMID: 38241304 PMC10798535

[B36] Tian L. Sun Z. Zhang Z. (2018). Antimicrobial resistance of pathogens causing nosocomial bloodstream infection in Hubei Province, China, from 2014 to 2016: a multicenter retrospective study. BMC Public Health 18, 1121. doi: 10.1186/s12889-018-6013-5, PMID: 30219056 PMC6138887

[B37] Wang C. Hao W. Yu R. Wang X. Zhang J. Wang B. (2021). Analysis of pathogen distribution and its antimicrobial resistance in bloodstream infections in hospitalized children in east China 2015-2018. J. Trop. Pediatr. 67, fmaa077. doi: 10.1093/tropej/fmaa077, PMID: 33367870 PMC7948388

[B38] Wen S. Harris P. Forde B. Permana B. Chatfield M. Lau C.. (2024). Characterization of gram-negative bloodstream infections in hospitalized Australian children and their clinical outcomes. Clin. Infect. Dis. 79, 734–743. doi: 10.1093/cid/ciae341, PMID: 38917034 PMC11426278

[B39] Williams A. Coombs G. Bell J. Daley D. Mowlaboccus S. Bryant P.. (2024). Antimicrobial Resistance in Enterobacterales, Acinetobacter spp. and Pseudomonas aeruginosa Isolates From Bloodstream Infections in Australian Children 2013-2021. J. Pediatr. Infect. Dis. Soc. 13, 617–625. doi: 10.1093/jpids/piae111, PMID: 39460715

[B40] Williams A. Coombs G. Bell J. Daley D. Mowlaboccus S. Bryant P.. (2025). Antimicrobial Resistance in Staphylococcus aureus and Enterococcus spp. Isolates From Bloodstream Infections in Australian Children 2013-2021. J. Pediatr. Infect. Dis. Soc. 14, piae110. doi: 10.1093/jpids/piae110, PMID: 39468748

[B41] Wu R. Cui X. Pan R. Li N. Zhang Y. Shu J.. (2025). Pathogenic characterization and drug resistance of neonatal sepsis in China: a systematic review and meta-analysis. Eur. J. Clin. Microbiol. Infect. Dis. 44, 779–788. doi: 10.1007/s10096-025-05048-1, PMID: 39853642

[B42] Xu H. Wu N. Yu H. Wang C. Deng J. Wang H.. (2024). Bacterial epidemiology and antimicrobial resistance profiles of bloodstream infections caused by negative bacteria in children’s: A multicenter study in China, (2016-2022). Infection Drug resistance 17, 4101–4112. doi: 10.2147/idr.S473227, PMID: 39319036 PMC11421437

[B43] Xu A. Zheng B. Xu Y. Huang Z. Zhong N. Zhuo C. (2016). National epidemiology of carbapenem-resistant and extensively drug-resistant Gram-negative bacteria isolated from blood samples in China in 2013. Clin. Microbiol. infection 22 Suppl 1, S1–S8. doi: 10.1016/j.cmi.2015.09.015, PMID: 26846351

[B44] Yadav P. Yadav S. (2022). Progress in diagnosis and treatment of neonatal sepsis: A review article. JNMA 60, 318–324. doi: 10.31729/jnma.7324, PMID: 35633256 PMC9226748

[B45] Yang P. Chen Y. Jiang S. Shen P. Lu X. Xiao Y. (2018). Association between antibiotic consumption and the rate of carbapenem-resistant Gram-negative bacteria from China based on 153 tertiary hospitals data in 2014. Antimicrobial resistance infection control 7, 137. doi: 10.1186/s13756-018-0430-1, PMID: 30479750 PMC6245771

[B46] Yang X. Ren L. Gong M. Lu Y. Ding X. (2024). Impacts of COVID-19 pandemic on culture-proven sepsis in neonates. Front. Cell. infection Microbiol. 14. doi: 10.3389/fcimb.2024.1391929, PMID: 38903936 PMC11186981

[B47] Yang M. Xin L. Li H. Lu X. Pan X. Lei S.. (2023). Risk factors for bloodstream infection in paediatric haematopoietic stem cell transplantation: a systematic review and meta-analysis. J. Hosp. infection 139, 11–22. doi: 10.1016/j.jhin.2023.06.003, PMID: 37308062

[B48] Yang S. Xu H. Sun J. Sun S. (2019). Shifting trends and age distribution of ESKAPEEc resistance in bloodstream infection, Southwest China 2012-2017. Antimicrobial resistance infection control 8, 61. doi: 10.1186/s13756-019-0499-1, PMID: 30976388 PMC6441235

[B49] Yilmaz S. Akkoc G. Aslan Tuncay S. Parlak B. Canizli Erdemli P. Dizi Isik A.. (2025). Pediatric Gram-negative bloodstream infections: epidemiology, antibiotic resistance, clinical outcomes and factors affecting mortality, a single center retrospective study. J. infection developing countries 19, 238–247. doi: 10.3855/jidc.20258, PMID: 40063749

[B50] Yu Y. He X. Wan L. Yang Y. Chen P. (2022). Etiology, antimicrobial resistance, and risk factors of neonatal sepsis in China: a systematic review and meta-analysis from data of 30 years. J. maternal-fetal neonatal Med. 35, 7541–7550. doi: 10.1080/14767058.2021.1951217, PMID: 34470123

[B51] Zeng D. Debabov D. Hartsell T. Cano R. Adams S. Schuyler J.. (2016). Approved glycopeptide antibacterial drugs: mechanism of action and resistance. Cold Spring Harbor Perspect. Med. 6, a026989. doi: 10.1101/cshperspect.a026989, PMID: 27663982 PMC5131748

[B52] Zhang D. Hu S. Sun J. Zhang L. Dong H. Feng W.. (2019). Antibiotic consumption versus the prevalence of carbapenem-resistant Gram-negative bacteria at a tertiary hospital in China from 2011 to 2017. J. infection Public Health 12, 195–199. doi: 10.1016/j.jiph.2018.10.003, PMID: 30385238

[B53] Zhang Z. Sun Z. Tian L. (2022). Antimicrobial resistance among pathogens causing bloodstream infections: A multicenter surveillance report over 20 years, (1998-2017). Infection Drug resistance 15, 249–260. doi: 10.2147/idr.S344875, PMID: 35115793 PMC8800585

[B54] Zhen X. Lundborg C. Sun X. Hu X. Dong H. (2019). Economic burden of antibiotic resistance in ESKAPE organisms: a systematic review. Antimicrobial resistance infection control 8, 137. doi: 10.1186/s13756-019-0590-7, PMID: 31417673 PMC6692939

[B55] Zheng C. Chen Q. Pan S. Li Y. Zhong L. Zhang X.. (2023). Staphylococcus aureus bloodstream infection in a Chinese tertiary-care hospital: A single-center retrospective study. Chin. Med. J. 136, 1503–1505. doi: 10.1097/cm9.0000000000002699, PMID: 37192008 PMC10278688

[B56] Zhou Y. Zhou S. Peng J. Min L. Chen Q. Ke J. (2023). Bacterial distribution and drug resistance in blood samples of children in Jiangxi Region 2017-2021. Front. Cell. infection Microbiol. 13. doi: 10.3389/fcimb.2023.1163312, PMID: 37424793 PMC10324674

